# Rules for Making Good Wine

**Published:** 1860-11

**Authors:** 


### *Rules for Making Good Wine.*

We would call the attention of our wine-growing community to the following rules for the manufacture of wine, which are communicated to the *Vigneron*, of France, a journal expressly devoted to the wine-growing interest, by M. de Babo, the president of an agricultural society, and an extensive proprietor and wine-grower at Weinheim, in the Grand-duchy of Baden :

1. The grapes should not be gathered until they have arrived at complete maturity, that is to say, when they do not grow sweeter, in a sensible degree. If the weather is good, they may be allowed to hang some time after this, for the purpose of giving the watery parts of the fruit time to evaporate. This increases considerably the strength and sweetness of the wine. Black grapes intended for red wine should not be allowed to become too ripe, as, if they do, they injure the color of the wine.

2. The vessels should be clean, and, above all, should not have contained sour wine. Care should also be taken that nothing should be allowed to fall into the must, which might cause acidity during the fermentation.

3. The white grapes should be put in a tub and pressed as quickly as possible, with the stems on. If obliged to wait before pressing the must, it is best to take out, at least, a portion of the stems which it contains, so that it shall not taste of them. The must of weak and mucilaginous wines ought to be allowed to ferment some days, with the stems, so that the tannin which they contain, will assist in the precipitation of the mucilaginous matter. For good wines, the mash, or residuum, of the grape, should never be pressed, as the last juice which comes from the press usually contains a great deal of acid, and but little sugar.

4. For the sharp wines of inferior quality, and for sweet and mucilaginous wines, it is indispensable to put the must into open tubs, and to leave it there for several days. There forms during this time a layer or stratum of a dirty brown color, which contains a great part of the mucilage, yeast and acid rejected by the must, and which should be taken off with care every time it forms, so as to remove all those subtances which alter the taste of the wine, cause fermentations, and do a great deal of mischief.

5. Care should be taken not to put the must into casks which are dirty, or which have been fumed with sulphur.—There are some wine-growers who think that the fumes of sulphur applied to casks preserve the sweetness of wine, and there are ignorant purchasers who permit themselves to be cheated as to the quality of the wine, by the sugar which the unfinished fermentation has left in it without decomposing it. But the following summer these wines are found to be muddy, and ferment often with great force, become sour, and are often completely spoiled. The wine, then, should be placed in casks which have not been fumed, and no obstacle to fermentation should be opposed, nor should it be arrested by the fumes of sulphur. There is no exception to this rule, save for those autumns which are unusually warm, and which cause fears that the fermentation will be too strong. In such a case, the vessels may be fumed with sulphur.

6. The fermentation of red wine should be treated differently from that of white. The must of black grapes may remain twenty-four hours, with the stems mixed with it, so that the tannin contained in them may communicate itself with the must. At the end of that time, the stems and the seeds should be separated by means of a sieve, and the must should be poured into open vessels, which should be lightly covered during the fermentation. The temperature of the must during the fermentation should not allowed to exceed 15 deg. of Reaumur (65¾ deg. Fahrenheit), in order to prevent the spirit from escaping. Every three or four hours the fermenting mass should be stirred, so as to prevent it from souring.

7. At the end of fifteen or twenty days, when all action has ceased, and the skins have yielded their coloring matter to the must, it should be put under the press and strongly squeezed, so that all the coloring matter shall be extracted. The wine is then placed in casks not fumed; and if it is desired to increase the capacity for tannin, some of the seeds, which should be separated by a sieve from the mesh, should be added to it.

8. If the weather is cold, the openings to the cellars should be closed, so that the fermentation may meet with no interruption. Persons should never enter the cellars until they have been tested for carbonic acid by a light. The carbonic acid may be driven from the cellars by opening all the issues, by lighting a fire on the stairway, by throwing hot water into them, and by scattering freshly-slacked lime in them. During the fermentation the bung-hole should only be closed with vine leaves, or by a little bag filled with sand—the object being to prevent the air from entering at the same time that the carbonic acid is permitted to escape.

9. Towards Christmas the clarification of the wine is about completed, and the yeast, which has become insoluble during the fermentation, is precipitated. Four weeks after the commencement of the fermentation, the casks, which should not be quite filled up at first, become completely full.

10. The racking, or drawing off from the lees at Christmas is very important and necessary. There always remains in the wine, after the first fermentation, a certain quantity of soluble leaven, and if this is not scattered, and the wine still contains undecomposed sugar, the liquid will become turbid, it will ferment again, and possibly be spoiled. In the first racking, towards the commencement of the year, care should be taken to expose the wine as much as possible to contact with the air, in which case the oxygen of the atmosphere precipitates the insoluble leaven, and the liquid clarifies completely, so that the second racking may be retarded until the end of April, there being no further fear of fermentation.

11. The following autumn anotner racking should take place, after which the wine may be considered as completely made. In drawing off, great care should be taken not to mix the portion of the wine at the bottom of the cask, which is still turbid, with the clear part which is above. The turbid part should be placed in a separate vessel, and submitted to a new racking before it is added to the other.

The author of these rules closes by saying: "If our wine-growers will strictly observe these prescriptions, without permitting themselves to be turned aside by local usages, they will obtain beautiful and good wines." It is possible, however, that our peculiar climate may justly entitle the vine-growers of California to make some modifications of M. de Babo's rules.—*Amer. Druggists' Circular.*

